# Supplementary figures and images for: Quantifying geographic accessibility to improve efficiency of entomological monitoring
Source: PLoS Negl Trop Dis. 2020 Mar 23;14(3):e0008096. doi: 10.1371/journal.pntd.0008096 (PMC7117774; doi:10.1371/journal.pntd.0008096)

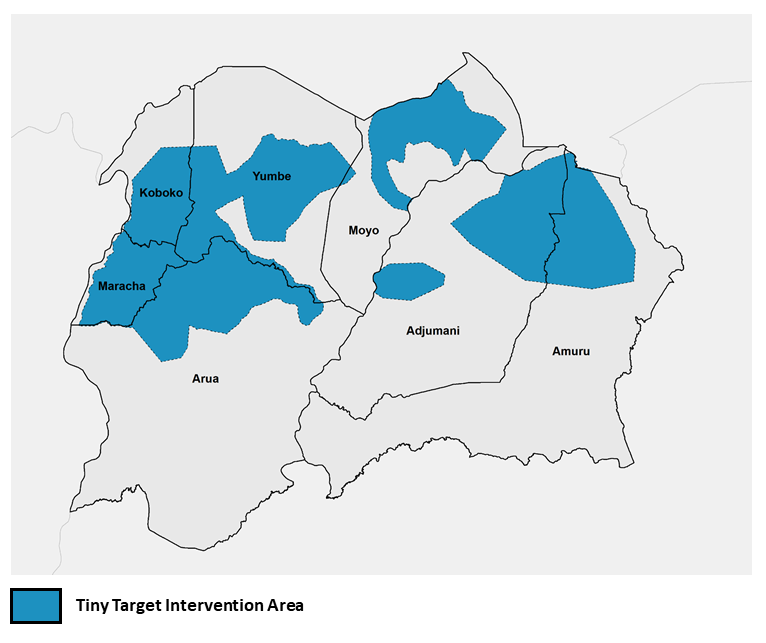

Supplement: S1 Fig — Blue areas identify both current and planned Tiny Target intervention areas within the West Nile Region of Northern Uganda. (TIF) [file pntd.0008096.s001.tif]

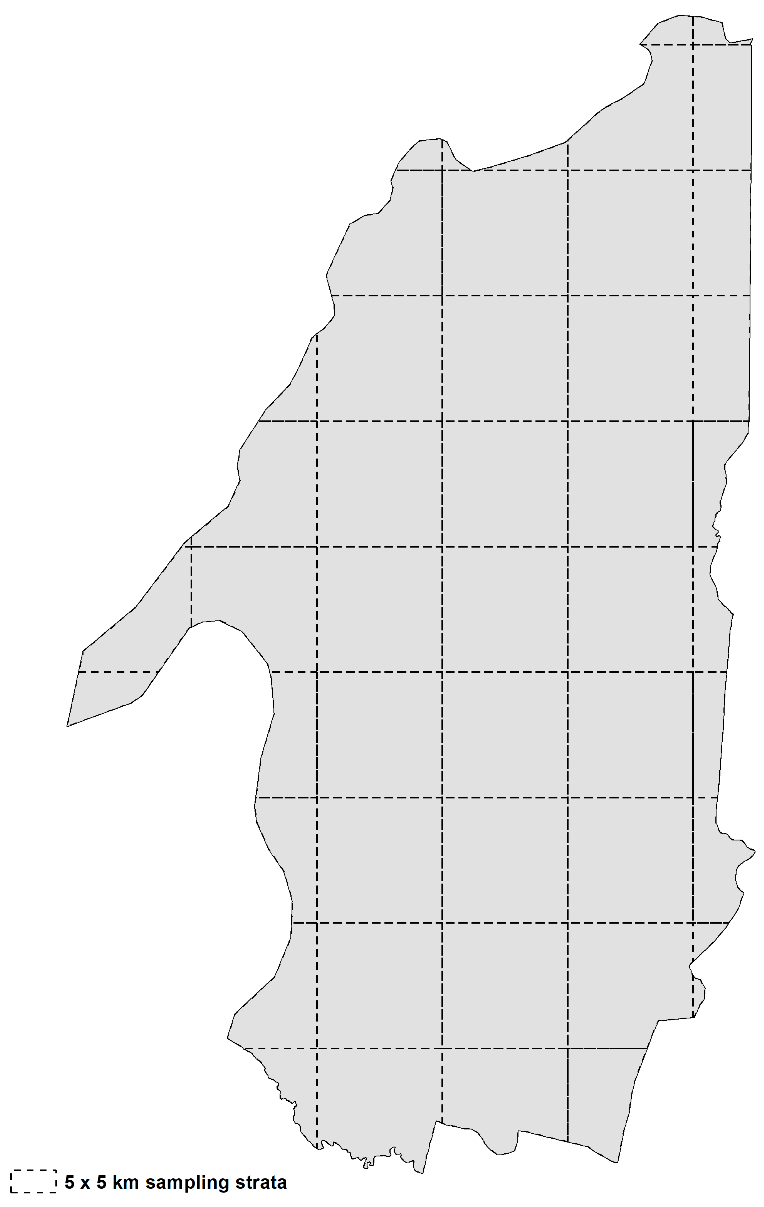

Supplement: S2 Fig — (TIF) [file pntd.0008096.s002.tif]

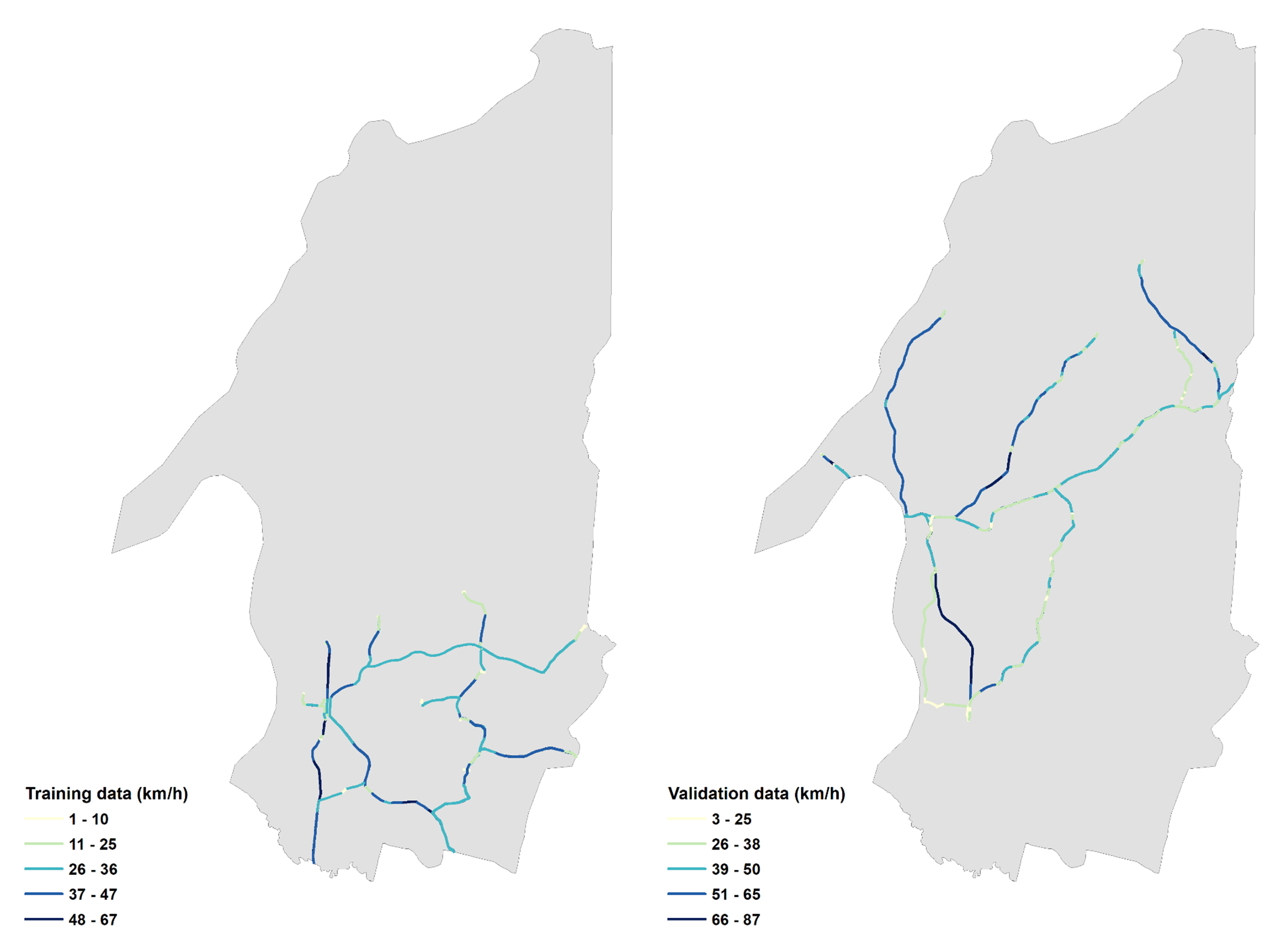

Supplement: S3 Fig — Left: Tracks used to inform speeds along select roads (training data). Right: Tracks used to validate the generated cost-distance surfaces (validation data). (TIF) [file pntd.0008096.s003.tif]

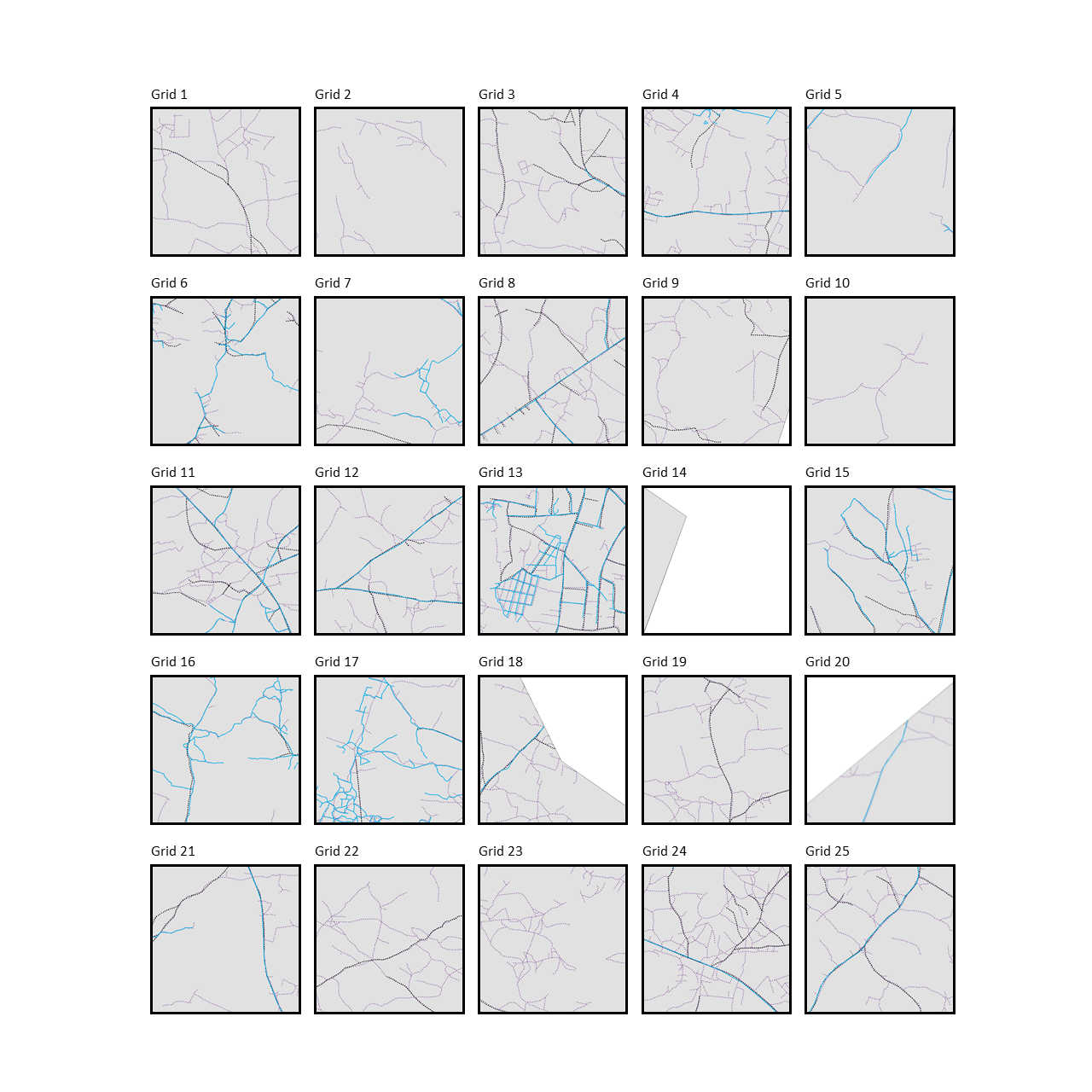

Supplement: S4 Fig — Purple roads represent roads visible in 0.5m imagery; black roads represent roads visible in 3m imagery, and light blue roads represent roads available within the OSM dataset. The overlap of all three colours indicate areas of consistency across sources. (TIF) [file pntd.0008096.s004.tif]

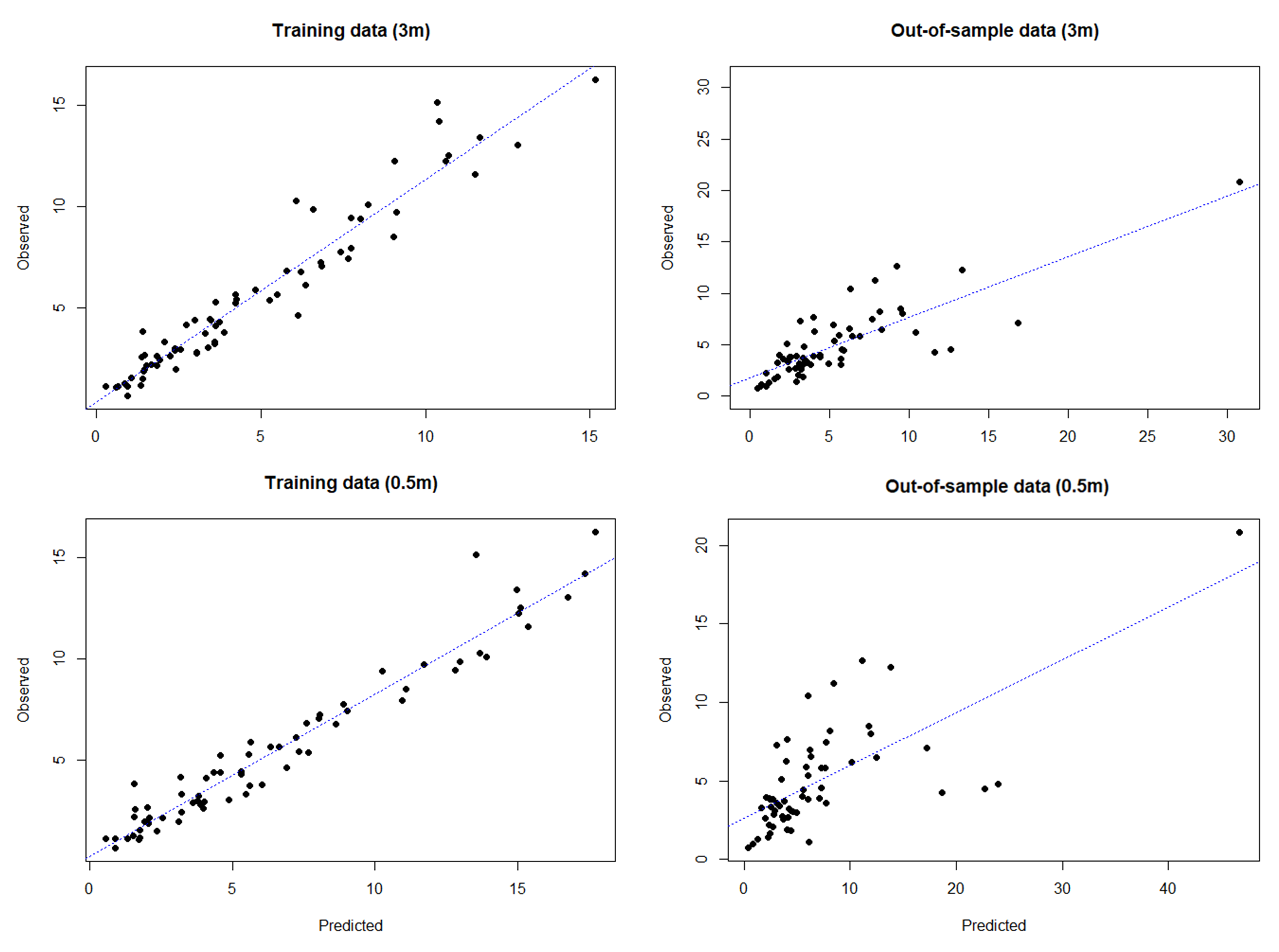

Supplement: S5 Fig — Plots from a linear regression using observed travel time data with predicted travel time as the only covariate. Top Left: Regression using 3m within-sample (training) data. Top Right: Regression using 3m out-of-sample (validation) data. Bottom Left: Regression using 0.5m within-sample (training) data. Bottom Right: Regression using 0.5m out-of-sample (validation) data. (TIF) [file pntd.0008096.s005.tif]
