# Supplementary material for: Quantifying geographic accessibility to improve efficiency of entomological monitoring
Source: PLoS Negl Trop Dis. 2020 Mar 23;14(3):e0008096. doi: 10.1371/journal.pntd.0008096 (PMC7117774; doi:10.1371/journal.pntd.0008096)
Supplement: S1 Table — (PDF) [file pntd.0008096.s006.pdf]

Table S1. Results of OpenStreetMap data validation.

Grid	Length of road digitized (m)			Percentage similarity (%)		
	0.5m	3m	OSM	OSM vs 0.5m	3m vs 0.5m	OSM vs 3m
1	7804.60	1459.33	0.00	0.00	18.70	0.00
2	3471.12	0.00	0.00	0.00	0.00	0.00
3	7759.08	3738.06	333.07	4.29	48.18	8.91
4	9146.50	1843.44	1817.94	19.88	20.15	98.62
5	2718.92	0.00	937.76	34.49	0.00	0.00
6	5515.98	2856.50	3483.76	63.16	51.79	121.96
7	3840.01	676.79	2254.52	58.71	17.62	333.12
8	10131.97	4607.29	1962.63	19.37	45.47	42.60
9	6053.58	1703.86	0.00	0.00	28.15	0.00
10	2034.04	0.00	0.00	0.00	0.00	0.00
11	12067.40	4540.82	3612.87	29.94	37.63	79.56
12	8940.11	2704.17	2082.35	23.29	30.25	77.01
13	14452.65	7053.10	9911.33	68.58	48.80	140.52
14	0.00	0.00	0.00	0.00	0.00	0.00
15	6650.08	3311.35	4432.14	66.65	49.79	133.85
16	3473.59	1370.35	5389.54	155.16	39.45	393.30
17	6779.36	772.51	7997.38	117.97	11.40	1035.25
18	5637.56	1214.75	649.64	11.52	21.55	53.48
19	9459.31	1912.29	0.00	0.00	20.22	0.00
20	2429.74	0.00	730.74	30.07	0.00	0.00
21	4846.82	2094.06	1343.11	27.71	43.20	64.14
22	8722.51	1564.75	0.00	0.00	17.94	0.00
23	8715.20	204.00	0.00	0.00	2.34	0.00
24	15891.30	4044.70	1064.19	6.70	25.45	26.31
25	8491.67	2918.87	1285.66	15.14	34.37	44.05
**Total**	**175033.09**	**50590.99**	**49288.63**	**28.16**	**28.90**	**97.43**
