# Supplementary material for: Quantifying geographic accessibility to improve efficiency of entomological monitoring
Source: PLoS Negl Trop Dis. 2020 Mar 23;14(3):e0008096. doi: 10.1371/journal.pntd.0008096 (PMC7117774; doi:10.1371/journal.pntd.0008096)
Supplement: S2 File — Available via FigShare: 10.6084/m9.figshare.11837070. (DOCX) [file pntd.0008096.s008.docx]

S2 File: 0.5 meter resolution resistance surface. Doi: 10.6084/m9.figshare.11837070 (<https://figshare.com/articles/0_5m_resolution_surface_-_Koboko/11837070>)
